# Establishment of an experimental model of ovalbumin-induced atopic dermatitis in canines

**DOI:** 10.3389/fvets.2024.1296138

**Published:** 2024-01-18

**Authors:** Ha-Young Shin, Hyung Jun Jin, Hyun-Jin Tae, Hong-Geun Oh, Jeong Ho Hwang

**Affiliations:** ^1^Animal Model Research Group, Korea Institute of Toxicology, Jeongeup, Republic of Korea; ^2^Companion Animal New Drug Development Center, Korea Institute of Toxicology, Jeongeup, Republic of Korea; ^3^College of Veterinary Medicine and Biosafety Research Institute, Jeonbuk National University, Iksan, Jeollabuk-do, Republic of Korea; ^4^R&D Division, HUVET Co., Ltd., Iksan-si, Republic of Korea

**Keywords:** ovalbumin, atopic dermatitis, canine atopic dermatitis, atopic dermatitis animal model, T helper 2 immune response

## Abstract

**Introduction:**

A reliable standard model is required to evaluate the efficacy of new drugs for companion animals, especially dogs. Canine atopic dermatitis (cAD), also known as allergic inflammatory skin disease, is a common condition. Currently, the house dust mite animal model is used in the research of cAD; however, this model exhibits significant individual variation and is difficult to standardize. In this study, we used ovalbumin as an antigen to sensitize and stimulate dogs, thereby establishing a stable model mimicking the T-helper 2 (Th2) response seen in cAD. Our objective was to create a cAD model that could be employed to evaluate the efficacy of novel drugs and mimic the Th2 dominant allergic response observed in the pathogenesis of atopic dermatitis of dogs.

**Methods:**

In this study, six beagles were used. Normal saline was applied to two animals, and ovalbumin to four, on their dorsal skin.

**Results:**

The ovalbumin-treated groups exhibited clinical cAD symptoms, such as pruritus and erythema. Moreover, plasma levels of the cAD markers immunoglobulin E and CCL17 chemokine were higher in the ovalbumin-treated group than in the vehicle control group. The skin thickness of the epidermis was significantly increased in the ovalbumin-treated group, with infiltration of inflammatory cells observed in the thickened dermis region. In conclusion, treatment of canine skin with an optimal concentration of ovalbumin induced typical cAD-like symptoms, and histological and molecular analyses confirmed an enhanced Th2-related immune response.

**Conclusion:**

Therefore, we successfully established a suitable Th2-dominant response mimicking cAD, which will facilitate targeted research of atopic dermatitis in dogs.

## 1 Introduction

Canine atopic dermatitis (cAD) is a common inflammatory skin disease that involves epidermal barrier dysfunction and recurrent pruritus in dogs (1). Atopic dermatitis (AD) is hereditary disease, although spontaneous development in any individual is possible. Environmental factors, such as exposure to allergens, play a significant role in triggering this condition (2). AD treatment in both humans and dogs involves utilizing medication that can suppress the T-helper 2 (Th2) cell response (3–6) and maintain long-term disease control (7–10).

Similar to humans, cAD skin lesions are characterized by the dermal infiltration of CD4^+^-T cells and eosinophils, known as the Th2 response. The Th2 response is an exaggerated immune reaction triggered by allergens (11). The immune system of genetically predisposed dogs recognizes allergens and activates Th2 cells. Activated Th2 cells migrate to the stimulation site via CCL17, a chemokine that moves inflammatory cells, and these cells stimulate B-cells (12). Th2 cells release specific signaling cytokines, including interleukin-4 (IL-4), interleukin-13 (IL-13), and interleukin-31 (IL-31). The released cytokines promote an inflammatory response in the skin, leading to characteristic symptoms of cAD, such as pruritus, erythema, and edema. The inflammatory T cell differentiation and Th2 immune response in the skin is regulated by inflammatory mediators, such as COX-2 (13, 14), but the role of COX-2 in the cAD immune response is unclear. Furthermore, the finding that inhibiting the COX-2 expression of an allergic atopic mouse model promotes a Th2 response (15) was an indicator for the construction of the Th2-dominant cAD model.

IL-4 and IL-13 mediate B cell immunoglobulin E (IgE) antibody hypersensitivity, which is involved in allergic reactions. IgE antibodies bind to mast cells, which are abundant in the skin. Upon subsequent exposure to the same allergen, IgE antibodies trigger mast cell degranulation. Degranulation results in the release of histamines and other mediators, leading to pruritus and subsequent inflammation. However, Th2 cytokines vary greatly depending on the sample and the time of measurement. In the case of CCL17, Th2-related chemokines can be measured in a relatively stable manner and, therefore, are being actively studied as targets that can determine the severity of allergic diseases such as AD.

Confirming the efficacy and safety of new drugs developed for treating diseases, including atopy, in target animals is essential to protect animals and ensure optimal treatment selection (16). Epicutaneous sensitization with allergens, such as house dust mites (HDMs), has been widely used to establish cAD models or to study contact allergy (17, 18). However, HDM-induced animals show highly individual variations, and standardization of protocols is difficult (19, 20). Therefore, reliable animal models are not available for conducting experiments, non-clinical studies, or clinical Phase II studies.

In this study, we used ovalbumin as an antigen for sensitization and stimulation to study its efficacy and immunological activity. We deduced the optimal concentration of ovalbumin necessary for modeling purposes and established a cAD model that mimics the Th2-dominant AD response. This model will serve as a valuable tool for further research in this field.

## 2 Materials and methods

### 2.1 Animals

Six male beagles (*Canis lupus familiaris*) were used in this experiment. The vehicle control (VC), T1, and T2 groups were composed of two animals each. All dogs were 7-month-old males with a mean weight of 7.95 kg (range, 7.2–8.7), and they were obtained from Raon Bio (Yongin, South Korea). Before the experiment, a 7-day acclimation period was followed by a veterinary quarantine. Healthy animals who did not develop dermatitis or allergic diseases during quarantine were included in this study. All the animals were housed in individual cages (120 × 180 × 150 cm^3^). During the experimental period, room temperature and humidity were maintained at 24°C (± 2°C) and 40–60%, respectively. Fluorescent light intensity was maintained at 150–300 lx, the air change rate was 10–15 times/h, and a 12-h light cycle was maintained. The animals were fed dry dog food (egg-free) and allowed to drink clean tap water voluntarily. Bathing was not performed during the study period to avoid changes in skin condition parameters. All animal experiments were conducted in accordance with the guidelines of the Institutional Animal Care and Use Committee of Huvet (IACUC approval number: HV 2022–011).

### 2.2 Study design

In this study, VC group were epicutaneously exposed to normal saline, T1 and T2 groups were epicutaneously exposed to 1 and 5 mg/kg of ovalbumin, respectively.

The study consisted of two phases. In Phase 1, the beagles were epicutaneously sensitized to ovalbumin for two weeks. At this time point, dogs were considered ovalbumin-sensitized even though most of them (3 out of 4) did not exhibit skin lesions. In Phase 2, the beagles were stimulated with ovalbumin for two weeks.

Ovalbumin was applied using Tegaderm™ transparent dressings (3 M, St. Paul, MN, United States). We decided to use ovalbumin on the dorsal area, which is not an area typical for developing cAD lesions because the animals were likely to remove the Tegaderm while moving. To create the cAD model, the dorsal skin of the experimental animals was clipped and sanitized with 70% isopropyl alcohol. The ovalbumin-treated groups were sensitized with 1 and 5 mg/kg of ovalbumin dissolved in normal saline every day for 2 weeks. Normal saline was administered to the VC animals. Macroscopic images were acquired on days 0, 2, 7, and 14.

### 2.3 Clinical evaluation of skin lesions

We assessed the severity of symptoms using a simplified version of the Draize scoring system used in toxicological and dermatological studies to evaluate skin irritation, with high scores indicating severe inflammation, injury, and swelling caused by ovalbumin. The Draize dermal irritation scoring system (DDISS) is scored twice a week (21), where 0 indicates no Erythema, Eschar, and Edema, 1 indicates barely noticeable Erythema, Eschar, and Edema, 2 indicates clearly visible Erythema, Eschar, and Edema, 3 indicates moderate to severe Erythema, Eschar, and Edema, and 4 indicates severe Erythema, and Eschar slightly deep tissue injuries, and severe Edema extending beyond the exposed area.

### 2.4 Sample collection and analysis

To analyze the complete blood count and blood chemistry, whole blood was collected in an anticoagulant ethylenediamine tetraacetic acid (EDTA) tube and a serum-separating tube (SST), respectively. A complete blood count analysis (BC-2800 Vet; Mindray, Shenzhen, China) was performed using whole blood samples. Blood samples in SSTs were centrifuged for 10 min at 3,000 rpm, and the supernatant was collected and used as serum for analysis (DRI-CHEM NX700; Fujifilm, Tokyo, Japan).

### 2.5 Histopathology

On day 15, all the animals were anesthetized to obtain skin samples at the dorsal regions. Four biopsies were collected using an 8 mm biopsy punch in the dorsal area from each of the six dogs after clinical evaluation and skin condition monitoring. For histopathological examination, 50% of the skin samples were fixed in 10% neutral-buffered formalin solution; for cytokine analysis, 50% of the samples preserved in RNA-later (AM7021, Invitrogen, Carlsbad, CA, United States) were instantly frozen in liquid nitrogen. Skin tissues were preserved in 10% neutral buffered formalin overnight and embedded in paraffin. Then, the tissue samples were sectioned at 5 μm intervals. Sectioned samples were deparaffinized and stained with hematoxylin and eosin to examine the structural abnormalities of the skin. Masson’s trichrome staining was performed according to the manufacturer’s instructions (Cat. No. IFU-2; ScyTek, Logan, UT, United States). The deparaffinized slides were incubated in Weigert’s iron hematoxylin solution, Biebrich scarlet-acid fuchsin solution, phosphotungstic–phosphomolybdic acid, aniline blue, and 1% acetic acid solution. The collagen in connective tissues was stained blue, the cytoplasm was stained red or pink, and the nuclei were stained dark red or purple. The staining results were examined by a histopathologist.

### 2.6 Quantitative reverse transcription-polymerase chain reaction

To measure the amount of ribonucleic acid (RNA) in the samples, RNA was extracted from skin biopsy tissues using the RNeasy Mini Kit (Qiagen, Hilden, Germany) and reverse transcribed using the QuantiNova Reverse Transcription Kit (Qiagen, Hilden, Germany). Reaction mixtures of volume 20 μL were prepared for qRT-PCR, which contained 2 × Power SYBR Green PCR Master Mix (Applied Biosystems, Waltham, MA, United States), and the reactions were performed using a QuantStudio 5 Real-Time PCR System (Applied Biosystems). Glyceraldehyde-3-phosphate dehydrogenase (GAPDH) was used as an endogenous control for normalization. Quantitative chain reactions were performed using intron-spanning primers, the sequences of which are listed in Table 1. Fold induction was quantified using the 2^−ΔΔCT^ method.

**Table 1:** Canine primers used for quantitative real-time PCR.

Gene symbol	Primer sequences (from 5′ to 3′)	Length	Gene Bank ID
*CCL17*	F: GCCATCGTGTTTGTAACT	103	XM_038657714.1
	R: CTCCCTTCCAGGTTCTTTGT		
*IL-4*	F: TCACCAGCACCTTTGTCCACGG	96	AF187322.1
	R: TGCACGAGTCGTTTCTCGCTGT		
*IL-13*	F: TGCGCAGCTCTAGAATCTCTG	69	NM_001003384.1
	R: CAGCATCCTCTGGGTCCTT		
*IL-31*	F: CCTGTTCCTGCTCTGCTCTA	188	NM_001165914.1
	R: TGAGACACAGCAGCAAGGTA		
*COX-2*	F: AAGCTTCGATTGACCAGAGCAG	145	NM_001003354.1
	R: TCACCATAAAGGGCCTCCAAC		
*PTGES*	F: GTCCTGGCGCTGGTGAGT	89	NM_001122854.1
	R: ATGACAGCCACCACGTACATCT		
*Filaggrin*	F: GATGACCCAGACACTGCTGA	158	XM_038423227.1
	R: TGGTTTTGCTCTGATGCTTG		
*GAPDH*	F: TTAACTCTGGCAAAGTGGATATTGT	85	XM_038448970.1
	R: GAATCATACTGGAACATGTACACCA		

### 2.7 Enzyme-linked immunosorbent assay

To analyze cytokines in the blood samples, whole blood samples were collected in anticoagulant EDTA tubes and centrifuged for 10 min at 3,000 rpm, and the supernatant was collected as plasma. All steps were conducted in the temperature range of 2–8°C. Canine IgE, IL-4, and IL-31 levels were measured using ELISA kits (Cat. No. CI0014 and CI0041 for IL-4 and IL-31, respectively; Neobiolab, Cambridge, MA, USA; Cat. No. ab157700 for IgE; Abcam, Cambridge, UK), according to the manufacturers’ protocols.

### 2.8 Statistical analysis

Prism 8 software (GraphPad Software, San Diego, CA, USA) was used for the statistical analysis. The mean ± standard deviation (SD) was used to represent the data in all graphs. For tissue analysis, at least three independent experiments were conducted in triplicate. For plasma analysis, blood was sampled from the cephalic vein of two animals per group, and all tests were performed in technical triplicate. Comparisons between groups were performed using the Student’s t-test. Statistical significance was set at a value of p of less than 0.05.

## 3 Results

### 3.1 Ovalbumin-sensitized dogs with clinical sympoms similar to typical cAD reactions

**Figure 1:**
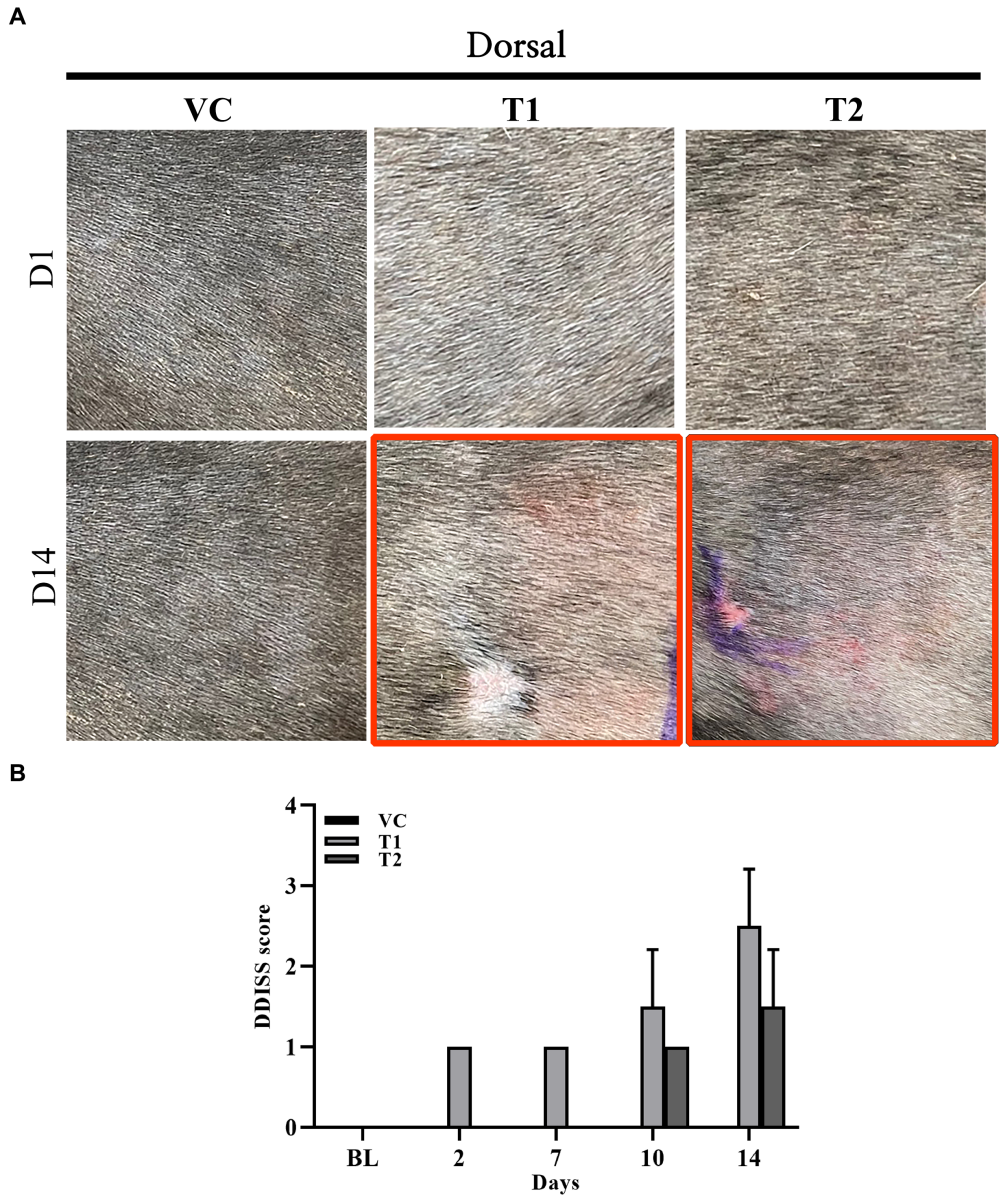
Changes in gross observations during ovalbumin stimulation. **(A)** Macroscopic image acquired on day 1 (top row) and 14 (bottom row) (gross observation) during ovalbumin stimulation in all groups (VC, T1, and T2). **(B)** Level of skin irritation scored using Draize dermal irritation scoring system (DDISS) in stimulation of ovalbumin. The ovalbumin-treated group exhibited more pronounced clinical symptoms of erythema formation compared to the vehicle control (VC) group. The scores were higher in the T1 (1 mg/mL) group compared to the T2 (5 mg/mL) group. The values represent mean ± SD. VC, vehicle control; T1, Treatment group 1 (low concentration ovalbumin treatment); T2, Treatment group 2 (high concentration ovalbumin treatment) *p < 0.05; **p < 0.01; ***p < 0.005.

The clinical symptoms of atopy in dogs include erythema, edema, and skin excoriations. We visually examined the typical clinical manifestations of atopy, including erythema, edema, and excoriations, in the ovalbumin-treated group. Symptoms were assessed and scored using the DDISS criteria. Compared to those of the VC group, the ovalbumin-treated group exhibited higher scores, with a notable increase observed in the T1 group compared to the T2 group. Furthermore, the severity score of dermatitis in the T1 group (Figures 1A,B).

### 3.2 Histopathology of ovalbumin-sensitized dogs similar to typical cAD

**Figure 2:**
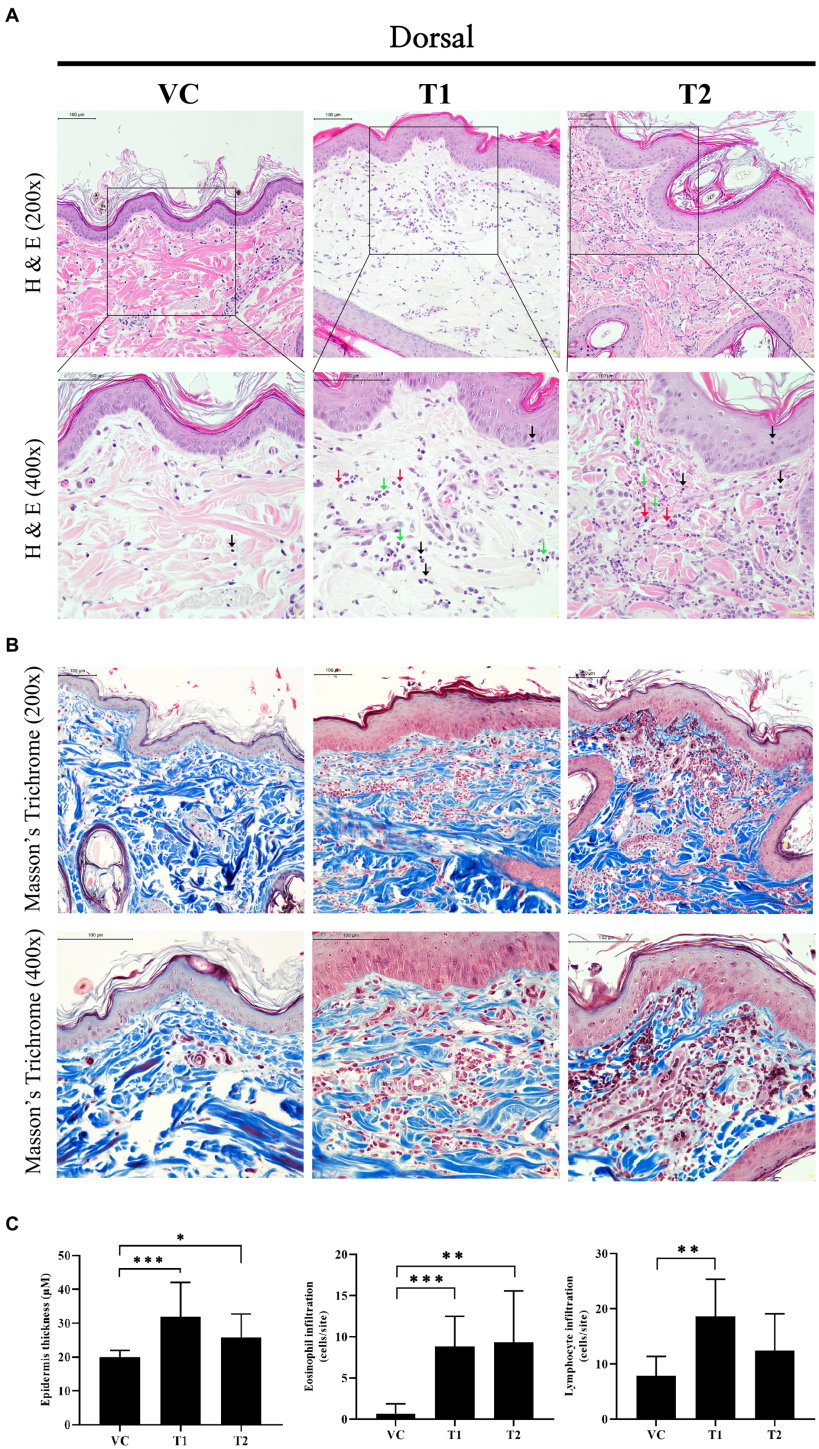
Changes in histopathology after ovalbumin stimulation. **(A)** Image showing histopathological analysis of hematoxylin and eosin staining in skin tissues. The top rows are 200x and the bottom row is 400x. Scale bar = 100 μm. Black arrow: Lymphocyte; Green arrow: Neutrophil; Red arrow: Eosinophil. Yellow star and arrow: structural abnormalities that lymphocytic dermal inflammation (perivascular inflammation) with focal epitheliotropism. **(B)** Image showing histopathological analysis of Masson’s trichrome staining in skin tissues. The top rows are 200x and the bottom row is 400x. Scale bar = 100 μm. **(C)** Epidermal thickness, and the number of infiltrating immune cells (Eosinophil, Lymphocyte). The values represent mean ± SD. VC, vehicle control; T1, Treatment group 1 (low concentration ovalbumin treatment); T2, Treatment group 2 (high concentration ovalbumin treatment) *p < 0.05; **p < 0.01; ***p < 0.005.

Histopathologically, cAD is characterized by a thickened epidermis resulting from an increased proliferation of keratinocytes, along with the infiltration of inflammatory cells, including lymphocytes and eosinophils. In our study, we noted a comparable infiltration of immune cells, including lymphocytes, eosinophils, and neutrophils, in the ovalbumin-treated group (Figures 2A,C). Additionally, we observed inflammatory cell infiltration and structural abnormalities that lymphocytic dermal inflammation (perivascular inflammation) with focal epitheliotropism in the dermal layer in the stratum corneum of the ovalbumin-treated group (Figures 2A,B). Histological examination using hematoxylin and eosin and Masson’s trichrome staining revealed a notable and statistically significant increase in the thickness of the epidermis in the group treated with ovalbumin compared to the VC group (Figure 2C). These findings indicate that the histopathological features of ovalbumin-induced AD closely mirror those commonly observed in patients with AD.

### 3.3 Ovalbumin-sensitized dogs similar to typical cAD downregulates Filaggrin But upregulates COX-2 expression and increases prostaglandin (PGE) production

**Figure 3:**
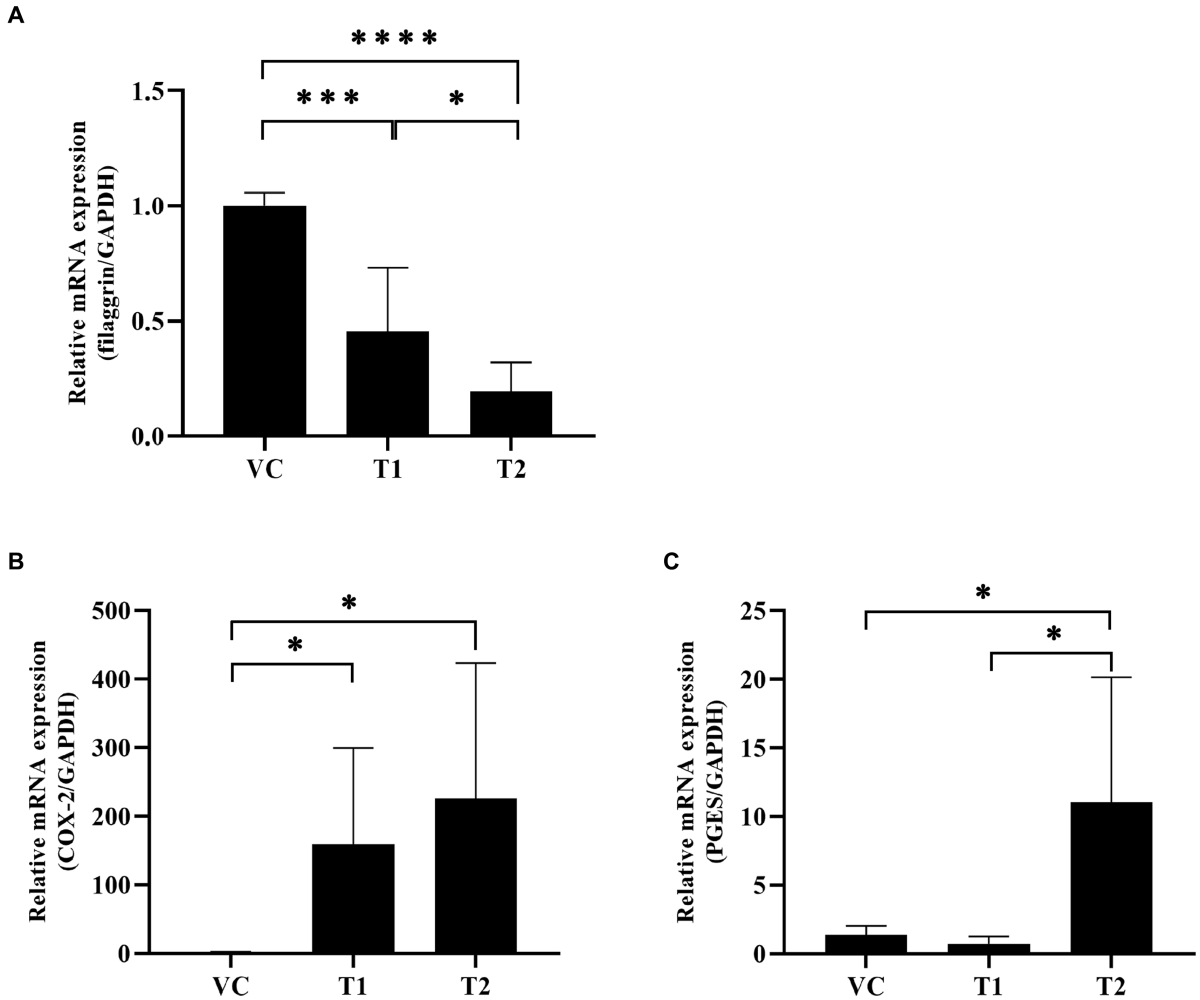
Changes in inflammation-related factors and skin barrier protein mRNA expression levels after ovalbumin stimulation. Levels of early inflammation-related cytokine mRNAs were quantified using reverse transcription-PCR. **(A)** The filaggrin mRNA expression level significantly decreased after ovalbumin stimulation. **(B)** The COX-2 mRNA expression level significantly increased after ovalbumin stimulation. **(C)** The PGES mRNA expression level significantly increased after ovalbumin stimulation T2 group, not T1 group. All values represent mean ± SD. VC, vehicle control; T1, Treatment group 1 (low concentration ovalbumin treatment); T2, Treatment group 2 (high concentration ovalbumin treatment) *p < 0.05; **p < 0.01; ***p < 0.005; ****p < 0.001. VC, vehicle control; PGES, prostaglandins.

In our cAD model, we examined the expression of key factors involved in the skin barrier, namely filaggrin and COX-2. Our mRNA analysis of the ovalbumin-exposed skin revealed a significant dose-dependent decrease in the expression levels of filaggrin in the ovalbumin-treated group compared to the VC group (Figure 3A). Additionally, ovalbumin treatment induced a significant increase in COX-2 expression and an associated increase in PGE2 production. These findings indicate that ovalbumin cause skin barrier damage and inflammation similar to cAD in a concentration-dependent manner (Figures 3B,C).

### 3.4 Ovalbumin-sensitized dogs results in a Th2-dominated response similar to typical cAD

**Figure 4:**
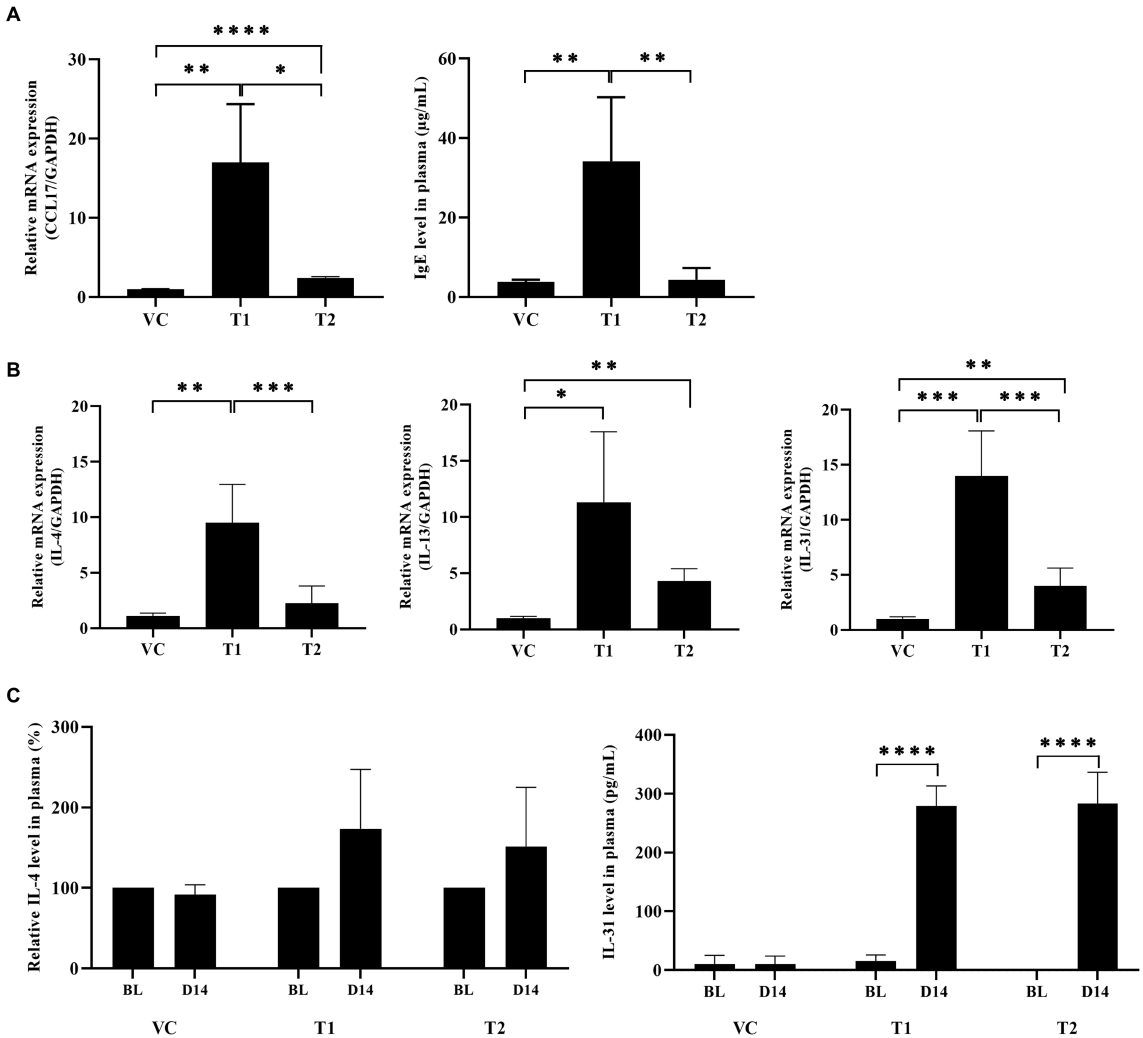
Ovalbumin induces a Th2-dominant response similar to typical cAD in ovalbumin-sensitized dogs. **(A)** Total IgE protein and CCL17 mRNA levels were upregulated in the ovalbumin-treated group compared to the VC group. **(B)** The mRNA expression levels of Th2-related cytokines in skin samples of cAD induced by ovalbumin were measured using qRT-PCR. **(C)** The protein expression levels of Th2-related cytokines in plasma samples obtained from individuals with AD were assessed using ELISA. All values represent mean ± SD. VC, vehicle control; T1, Treatment group 1 (low concentration ovalbumin treatment); T2, Treatment group 2 (high concentration ovalbumin treatment) *p < 0.05; **p < 0.01; ***p < 0.005; ****p < 0.001. VC, vehicle control.

CCL17, commonly known as thymus- and activation-regulated chemokine (TARC), is an important chemokine expressed in patients with AD. CCL17 stimulates CCL4^+^-Th2 cell migration to the site of allergen exposure (22). In cAD, upregulation of Th2 cell-related cytokine expression is observed not only in the skin, but also in peripheral blood mononuclear cells (PBMCs) and plasma samples. In contrast, IgE generates allergen-specific antibodies that bind to mast cells and trigger the release of inflammatory molecules. Consequently, we identified two factors that play a role in the initial stage of the AD response. Our findings demonstrated a significant upregulation of CCL17 chemokine expression at the mRNA level in the ovalbumin-treated group compared to the VC group. Additionally, we assessed IgE levels to confirm the production of serum total IgE antibodies in ovalbumin-induced AD. Interestingly, both CCL17 expression and IgE levels were higher in the low-dose group than in the high-dose group (Figure 4A). Furthermore, we analyzed the cytokines secreted by the T cells attracted to the site of ovalbumin treatment. This analysis was performed using skin and plasma samples, which confirmed the presence of Th2 cells recruited into the skin. We also observed the predominant secretion of cytokines associated with Th2 cells, including IL-4, IL-13, and IL-31, in the skin. The mRNA levels of these cytokines showed a significant increase in the ovalbumin-treated group compared with the VC group (Figure 4B). No Th1 expression was detected in the ovalbumin-treated group. Furthermore, we observed a significant elevation in IL-4 and IL-31 levels in plasma samples from the OVA-treated group. This observation was more pronounced in the low-dose group than in the high-dose group (Figure 4C).

## 4 Discussion

In this study, we aimed to establish a Th2-dominant response cAD model using ovalbumin in healthy beagle dogs with no known history of genetic predisposition for developing cAD. In the sensitization and induction stages, using Tegaderm, ovalbumin was applied daily to dogs without bathing. The clinical symptoms of atopy were analyzed twice a week using visual scoring. Subsequently, histological and immunological analyses were conducted after the ovalbumin application period to assess the AD model using skin exposed to ovalbumin.

Atopic dermatitis is a common inflammatory skin condition affecting both humans and dogs globally (23, 24). Treatment for human atopic dermatitis and cAD aims to relieve symptoms such as pruritus and inflammation through various pharmacological interventions. Nevertheless, the efficacy has not been extensively studied in dog. Thus, additional targeted research is needed. Consequently, a reliable cAD model is needed for research purposes.

In a majority of dogs with cAD an increased level of circulating IgE toward environmental allergens can be seen. Therefore, it may be common practice to detect levels of IgE specific to common allergens in most dogs diagnosed with AD. The primary method used to induce AD like symptoms in dogs for research purposes is the application of HDMs (20, 25). Some studies have screened dogs for high IgE before inducing AD (19, 25). In some studies, healthy beagle dogs without a genetic predisposition to elevated IgE levels were used; however, the evaluation relied on IgE as the sole assessment index. Chemokines like CCL17, which are elevated in allergic AD, facilitate the recruitment of Th2 cells to inflammation sites, and the Th2 cytokine (IL4) stimulates IgE synthesis (26). Consequently, given that IgE levels in atopic dermatitis correlate with a Th2 immune response, it becomes important to examine the chemokines or cytokines that are regulated in conjunction with IgE levels.

Therefore, the establishment of standardized HDM-induced atopy models for research purposes is challenging. Moreover, increased IgE levels serve as markers of allergen in AD. However, an elevated IgE level is an important indicator that cannot be solely relied upon for the definitive diagnosis of AD (1). In both mice and minipigs, AD like symptoms can be induced via the dermal application of ovalbumin (27–29). In ovalbumin-induced AD mouse models, the induction of atopy through percutaneous sensitization compromises the skin barrier function, enabling allergens to penetrate the skin. Subsequently, repeated transdermal sensitization triggers a Th2 response, resulting in the manifestation of cutaneous symptoms. Consequently, we established a cAD model by utilizing ovalbumin as a transdermal sensitizer. Furthermore, our findings suggest that elevated IgE levels, accompanied by CCL17 chemokine and Th2-dominant responses, play a significant role in the exacerbation of ovalbumin-induced AD. Atopic dermatitis in dogs is accompanied by macular erythema, rash, and pruritus (30, 31). Moreover, any compromise in skin barrier function contributes to the accelerated progression of AD. When the skin encounters allergens, there is an increase in inflammatory mediators like PGEs and COX-2. Regarding the Th1/Th2 imbalance, topical *in vitro* application of PGE2 suppresses IL-12 cytokine production, which is essential for releasing IFNg, a Th1 cytokine (32). Inhibition of IL-12 promotes a Th2 immune response (33), leading to rapid skin irritation development (34). Interestingly, previous research demonstrated that selectively inhibiting COX-2 expression in a mouse model of allergic atopy favored a Th2 response, exacerbating AD symptoms (15). In our study, the low ovalbumin exposure group (T1 group) exhibited decreased expression of COX-2 and PGE compared to the higher exposure group (T2 group). This suggests that reduced ovalbumin exposure may lead to a diminished COX-2/PGE regulatory mechanism, subsequently increasing specific Th2 cytokine expressions. Therefore, we propose that the optimal concentration for inducing a Th2-dominant cAD model is 1 mg/mL. However, this hypothesis requires further verification in future studies involving larger numbers of dogs.

AD may be associated with a deficiency of filaggrin, a key protein involved in the formation of the outermost layer of the skin (stratum corneum), in some individuals. The Th2 polarized immune response induced by acute AD leads to a secondary decrease in filaggrin. Therefore, the decreased in filaggrin may exacerbate the dermal inflammatory disease and need to be confirmed in association with Th2 cytokine in AD (35–37). Our findings demonstrated that the pattern of change in expression levels of filaggrin was similar to that observed in earlier studies (38, 39) In the ovalbumin-treated group, a concentration-dependent decrease in filaggrin mRNA expression levels was observed. This indicates that the application of ovalbumin impaired skin barrier function, which resembles the effects observed in patients with atopy.

During the initial phases of allergen penetration caused by skin barrier deficiencies, the chemokine CCL17 is released by skin cells, such as keratinocytes, dendritic cells, and endothelial cells. Subsequently, CCL17 is expressed by CCR4^+^-Th2 cells in the late stages of allergen penetration. In a study conducted by Kakinuma et al., individuals with AD had elevated serum levels of CCL17 compared to patients with psoriasis. This finding highlights the significant role of CCL17, an essential chemokine, in the progression and development of AD (40). In our study, we observed a substantial increase in the mRNA expression levels of CCL17 in the ovalbumin-induced AD group compared to the control group. Interestingly, this increase was particularly prominent in the low-dose group. This suggests that the level of CCL17 increased by ovalbumin led to a Th2-dominant immune response in the acute phase and that 1 mg/mL was the appropriate concentration to induce a Th2-dominant immune response.

The histopathological findings of our study revealed the infiltration of immune cell (lymphocyte, eosinophil, and neutrophil) in all groups analyzed. Notably, a significant increase in the number of infiltrating eosinophil and lymphocyte cells were observed in the ovalbumin-treated group (Figure 2B). The infiltration of inflammatory cells into skin lesions in AD is typically characterized by the presence of various subpopulations of T cells and type 2 innate lymphoid cells (41). Nakatani et al. reported a higher incidence of CCR4^+^-CD4^+^-T cell infiltration in patients with chronic AD compared to patients with psoriasis (42). Additionally, Murra et al. reported that the administration of a CCR4 antagonist in a cAD model resulted in the effective suppression of clinical symptoms associated with cAD (43). Our research revealed a consistent correlation between augmented eosinophilic and lymphocytic dermal inflammation (perivascular inflammation) infiltration of immune cells in ovalbumin-induced AD, elevated expression levels of CCL17 mRNA, and an increased presence of Th2-dominant response cytokines.

AD is primarily characterized by the dominance of the Th2 immune response, leading to the upregulation of cytokines, such as IL-4, IL-5, and IL-13 (44–46). The topical application of ovalbumin in mouse models results in the upregulation of Th2-related chemokines, such as CCL17 and CCL22, promoting the infiltration of Th2 cells and intensifying the allergic inflammatory response (47). Assessment of plasma and skin tissues of the ovalbumin-treated group revealed a significant elevation in IL-4 protein and mRNA levels, as well as IL-13 mRNA levels. Interestingly, our findings revealed that the expression levels were notably high in the low-dose group.

IL-31 inflammatory cytokine is produced by Th2 cell and is directly involved in activating sensory neurons and triggering skin pruritus (48, 49). In individuals with AD, there was a notable increase in IL-31 levels compared to healthy individuals. In addition, IL-31 injection in dogs induced pruritus (50). Our findings revealed a substantial increase in IL-31 protein and mRNA expression levels in the ovalbumin-treated group. Therefore, we determined the optimal concentration of ovalbumin required for inducing atopy. We also observed that the symptoms of AD induced using the optimal concentration of ovalbumin closely resembled the clinical symptoms observed in patients with AD, and this finding validates the relevance and importance of our model for conducting studies on AD.

There are three main limitations to this study. The first limitation is that all groups comprised two dogs, which may introduce a bias that could affect the statistical analysis. However, this limitation was resolved with four biopsies of the dorsal region treated with Ovalbumin taken from each dog and estimated with at least three replicates of the analysis. Second, the CADESI-04, a clinical assessment scale for canine atopy, was not applicable to our study. Our cAD model, with ovalbumin applied locally to the back rather than systemically sensitized AD, is a model for studies in which a Th2-dominant immune response is observed in the back with clinical manifestations similar to atopic dermatitis. However, the dorsum is not a common site of atopic dermatitis and is therefore not included in the CADESI-04 metrics. We needed a metric to assess clinical symptoms and applied the DDISS, which we used in our previous minipig AD model study (29). The graph we presented scores the atopic clinical manifestations of erythema and edema. In our study, eschar formation was consistently scored as 0 for all animals. In the future, we will apply indicators that can reflect the clinical manifestations of atopic dermatitis (erythema, edema, excoriation, alopecia) as a modified skin response score or a modified CADESI score, where lesion type and skin severity relevant for this model is assessed. A third limitation is that itching in this model was based on casual visual observation by the veterinarian. Although scales are available to determine clinical pruritus, the scales are designed for dog owners (51). Pruritus is a sporadic symptom and we were unable to control for it in our study. Therefore, pruritus scales should be further developed for future researchers.

When canine skin was treated with an optimal concentration of ovalbumin, we observed clinical, histopathological, and non-clinical changes that resembled those observed in naturally occurring cAD. Additionally, we observed an enhanced Th2-related immune response. In summary, we established a cAD animal model that can be used for basic Th2-dominant response research in the realm of cAD, which provides a means for accurate assessment of the safety of novel drugs for the treatment of cAD.

## Data availability statement

The original contributions presented in the study are included in the article/supplementary material, further inquiries can be directed to the corresponding author.

## Ethics statement

The animal study was reviewed and approved by all animal experiments were conducted in accordance with the guidelines of the Institutional Animal Care and Use Committee of Huvet (IACUC approval number: HV 2022-011).

## Author contributions

H-YS: Formal analysis, Methodology, Visualization, Writing – original draft, Writing – review & editing. HJ: Methodology, Investigation. H-JT: Methodology, Validation, Writing – review & editing. H-GO: Supervision, Writing – original draft. JH: Conceptualization, Funding acquisition, Supervision, Resources, Writing – review & editing.
